# A Pilot Study on Linking Tissue Mechanics with Load-Dependent Collagen Microstructures in Porcine Tricuspid Valve Leaflets

**DOI:** 10.3390/bioengineering7020060

**Published:** 2020-06-18

**Authors:** Luke T. Hudson, Samuel V. Jett, Katherine E. Kramer, Devin W. Laurence, Colton J. Ross, Rheal A. Towner, Ryan Baumwart, Ki Moo Lim, Arshid Mir, Harold M. Burkhart, Yi Wu, Chung-Hao Lee

**Affiliations:** 1 Biomechanics and Biomaterials Design Laboratory (BBDL), School of Aerospace and Mechanical Engineering, The University of Oklahoma, Norman, OK 73019, USA; luke.t.hudson-1@ou.edu (L.T.H.); samuel.v.jett-1@ou.edu (S.V.J.); Katherine.E.Kramer-1@ou.edu (K.E.K.); dwlaur@ou.edu (D.W.L.); cjross@ou.edu (C.J.R.); yiwu@ou.edu (Y.W.); 2 Advanced Magnetic Resonance Center, MS 60, Oklahoma Medical Research Foundation, Oklahoma City, OK 73104, USA; Rheal-Towner@omrf.org; 3 Department of Veterinary Clinical Sciences, College of Veterinary Medicine, Washington State University, Pullman, WA 99164, USA; ryan.baumwart@wsu.edu; 4 Department of Medical IT Convergence Engineering, Kumoh National Institute of Technology, Gumi, Gyeongbuk 39177, Korea; kmlim@kumoh.ac.kr; 5 Division of Pediatric Cardiology, Department of Pediatrics, The University of Oklahoma Health Sciences Center, Oklahoma City, OK 73104, USA; Arshid-Mir@ouhsc.edu; 6 Division of Cardiothoracic Surgery, Department of Surgery, The University of Oklahoma Health Sciences Center, Oklahoma City, OK 73104, USA; Harold-Burkhart@ouhsc.edu; 7 Institute for Biomedical Engineering, Science and Technology (IBEST), The University of Oklahoma, Norman, OK 73019, USA

**Keywords:** tricuspid regurgitation, biaxial mechanical testing, polarized spatial frequency domain imaging, spatial alignment, collagen fiber reorientation, material anisotropy

## Abstract

The tricuspid valve (TV) is composed of three leaflets that coapt during systole to prevent deoxygenated blood from re-entering the right atrium. The connection between the TV leaflets’ microstructure and the tissue-level mechanical responses has yet to be fully understood in the TV biomechanics society. This pilot study sought to examine the load-dependent collagen fiber architecture of the three TV leaflets, by employing a multiscale, combined experimental approach that utilizes tissue-level biaxial mechanical characterizations, micro-level collagen fiber quantification, and histological analysis. Our results showed that the three TV leaflets displayed greater extensibility in the tissues’ radial direction than in the circumferential direction, consistently under different applied biaxial tensions. Additionally, collagen fibers reoriented towards the direction of the larger applied load, with the largest changes in the alignment of the collagen fibers under radially-dominant loading. Moreover, collagen fibers in the belly region of the TV leaflets were found to experience greater reorientations compared to the tissue region closer to the TV annulus. Furthermore, histological examinations of the TV leaflets displayed significant regional variation in constituent mass fraction, highlighting the heterogeneous collagen microstructure. The combined experimental approach presented in this work enables the connection of tissue mechanics, collagen fiber microstructure, and morphology for the TV leaflets. This experimental methodology also provides a new research platform for future developments, such as multiscale models for the TVs, and the design of bioprosthetic heart valves that could better mimic the mechanical, microstructural, and morphological characteristics of the native tricuspid valve leaflets.

## 1. Introduction

The tricuspid valve (TV) plays a key role in regulating the unidirectional blood flow within the right side of the heart. The TV is composed of three collagenous leaflets that are attached to the right atrium at the valvular annulus and connected to the papillary muscles of the right ventricular walls through the chordae tendineae. Proper opening and closing of the TV leaflets prevent the backflow of deoxygenated blood from the right ventricle (RV) into the right atrium. Pressure and/or volume overload in the RV can cause alterations in the right ventricular geometry and further result in the development of diseases such as functional tricuspid regurgitation (FTR). These organ-level changes are prevalent among the general population and can be detrimental, potentially leading to heart failure [1,2,3]. Despite these repercussions, FTR has been largely under-investigated in the last two decades compared to other valvular heart diseases (e.g., left-sided heart valves: the mitral valve and the aortic valve). This may originate from the pervasive expectation among surgeons and cardiologists that correcting those left-sided cardiac lesions will naturally resolve the accompanied FTR [4]. However, recent studies by Dreyfus et al. (2005) and Anyanwu and Adams (2010) [2,5] have shown that this conservative clinical viewpoint and practice may not be valid, and those untreated FTR cases later progress to severe TR that further worsens long-term prognosis.

Apart from these organ-level, geometrical changes, tissue remodeling has been observed on the microscopic level, indicating the importance of the leaflets’ microstructure in connection to proper physiological function [6,7,8]. The microstructure of the TV leaflets can be morphologically categorized into four distinct layers, as described from the atrial to the ventricular surfaces [9,10,11]: (i) the atrialis, composed primarily of radially-oriented elastin fibers that provide the tissues’ low-stress elasticity; (ii) the spongiosa, containing non-fibrous components, such as glycosaminoglycans (GAGs) and proteoglycans (PGs), that act as a dampening mechanism during rapid leaflet bending; (iii) the collagen-rich fibrosa—the primary load bearing layer; and (iv) the ventricularis, which is composed of circumferentially-oriented elastin fibers that facilitate movement and restore the leaflets to their undeformed geometries during TV opening [12,13]. The connection of the tissue-level and microstructural changes to the overall organ-level TV function have not yet been fully understood in the heart valve biomechanics society. Quantifying these microstructural and tissue-level changes can inform accurate constitutive models, that may be used in in-silico finite element simulations for guiding clinical therapeutics.

In spite of the lack of connections between the microstructures and tissue biomechanics to the organ-level TV function, research efforts have been made in the past 3–5 years to characterize the tissue mechanics of the TV leaflets [10,14,15,16,17] and other sub-valvular components, such as the chordae tendineae [18,19,20] and the TV annulus [21,22,23,24,25,26], in addition to evolving clinical studies on the TV function [27,28] and post-operative outcome [29,30,31]. Previous studies of the heart valve leaflets have shown that the collagen fiber networks adjust and reorient in response to applied mechanical loading [32,33,34,35]. More recently, Laurence et al. (2019) further investigated the regional variations in the biaxial mechanical and biaxial stress relaxation properties of the TV leaflets [36]. The variance between the central (belly) region and the edge region (closer to the commissure) elicits the need for a more in-depth understanding of the microstructural differences within the TV leaflet tissue, that would aid in establishing such a connection to the organ-level TV function. In addition, the mechanical properties of specific tissue layers were also examined by Kramer et al. (2019) [9]. Specifically, in the TV anterior leaflet, different tissue constituents may contribute to the different mechanical behaviors of each layer (i.e., a more compliant stress-strain response in the combined atrialis/spongiosa layer, as opposed to the combined fibrosa/ventricularis layer). Evidently, the role of the collagen fiber architecture in tissue mechanics warrants further investigations to improve our understanding of current TV pathologies and function.

On the other hand, to examine collagen fiber networks, optical techniques, such as second harmonic generation (SHG) imaging [35,37,38,39] and small angle light scattering (SALS) [40,41], have been employed to provide valuable insight into the microstructural characterization of the heart valve leaflets and other soft tissues. However, these techniques are limited in their ability to capture the spatially varied, load-dependent collagen fiber architecture. For example, SHG has been used to obtain high-resolution images of the collagen fiber architecture of the mitral valve anterior leaflet at a biaxially-loaded state [34]. This investigation was limited to a micron-level field of view (FOV), restricting the SHG modality from effectively examining the spatial variance in the collagen fiber architectures (CFAs) at a larger FOV. In contrast, the SALS imaging modality can capture the CFA of the entire tissue specimen, but this technique requires chemical fixation along with optical clearing solution [42,43], limiting its capability to investigate the adaptive, load-dependent collagen fiber architecture. To addresses these shortcomings, polarized spatial frequency domain imaging (pSDFI), a recently developed optical imaging technique, has been used to observe the CFA of the tissue on a millimeter-scale FOV, while removing the need for fixative solutions. Previously, Goth et al. [44] displayed the collagen microstructural quantification capabilities of pSFDI using ovine aortic heart valve leaflets at an unloaded state; however, they did not yet highlight the load-dependent architectural changes. Recently, our group has developed a combined instrument, which integrated an in-house pSFDI device with a commercial biaxial testing system, to investigate the load-dependent changes in the CFAs for bovine tendon tissues, with highly-aligned CFAs, and a representative porcine mitral valve anterior leaflet with more dispersed CFAs [45].

Thus, the objective of this pilot study is to utilize the above combined instrument to observe the load-dependent changes in the CFAs for three TV leaflets from a representative porcine heart. This investigation will provide key insight into the microstructures and tissue-level mechanics of the TV. The observed load-dependent changes in the CFAs at the microstructural level will aid in providing a better understanding of how the TV diseases influence the tissue mechanics and overall TV function, and in informing TV computational models for guiding clinical therapeutics, such as feasibility or valve repair.

## 2. Materials and Methods

### 2.1. Porcine Heart Acquisition and Tissue Preparation

**Figure 1:**
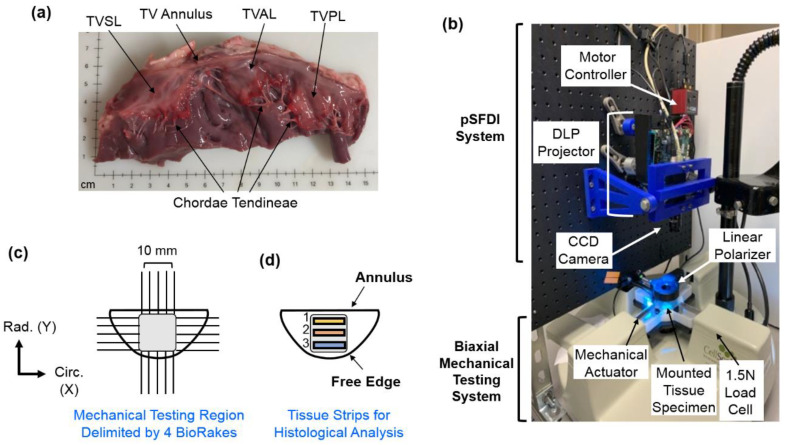
(**a**) The porcine TV was dissected to retrieve the three TV leaflets for use in (**b**) biaxial mechanical testing. (**c**) A side view of the combined pSFDI-biaxial testing system, displaying optical components, mounted tissue specimen, and mechanical testing components. (**d**) TV leaflet strips from the effective testing region were used for histological analyses.

Three fresh, normal porcine hearts were obtained from a local USDA-approved abattoir (Country Home Meat Company, Edmond, OK, USA), and dissected to retrieve the three TV leaflets (Figure 1a): the anterior leaflet (TVAL), the posterior leaflet (TVPL), and the septal leaflet (TVSL) (*n* = 3 for each of the three TV leaflets). Excess chordae tissue was trimmed from the leaflets, and thickness measurements were made using a digital caliper (Westward Tools 1AAU4—0.01 mm resolution) at three different locations, to determine an average leaflet thickness. Tissues were then stored in phosphate-buffered saline (PBS) at 4 °C, prior to testing within 48 h.

### 2.2. Biaxial Mechanical Testing

The TV leaflet tissues were mounted to a commercial biaxial testing system (CellScale, Canada, 1.5 N load cells), using four BioRakes to facilitate a 10 × 10 mm effective testing region in the central portion of the leaflet specimens (Figure 1b,c). During mounting, the tissues’ circumferential (*C*) and radial (*R*) directions were aligned with the *x*- and *y*-directions of the biaxial testing device. Following our previously developed displacement-controlled protocols [9,10,36,46,47] for investigating the atrioventricular heart valve tissue’s nonlinear mechanical behaviors and material anisotropy, the specimen was next submerged in a PBS bath at 37 °C for the entire duration of mechanical testing. A preconditioning protocol was utilized with six equibiaxial and non-equibiaxial loading/unloading cycles, targeting a membrane tension of 25 N/m to restore the leaflets to their in-vivo physiological configurations [14,48]. After preconditioning, the tissues were subjected to displacement-controlled biaxial testing experiments, emulating biaxial tensions (*T*) at varying loading ratios (*T_C_*:*T_R_* = 1:1, 1:0.5, 0.5:1, and 2:2), to capture a broad range of potential physiological deformation states [10,17,19]. These biaxial tension protocols were each applied for three loading/unloading cycles, and the forces and displacements were recorded at 5 Hz. The membrane tension-stretch data was extracted from the third loading cycle. Note that the membrane tension was computed from the recorded force divided by the effective edge length (10 mm), and the tissue stretch (*λ*) was calculated using $d_{tine}^{load}/d_{tine}^{PPC}$ for both the circumferential and radial directions, where $d_{tine}^{PPC}$ is the tine distance after preconditioning, and $d_{tine}^{load}$ is the tine-to-tine distance at the loading state.

### 2.3. pSFDI-Based Collagen Microstructure Quantifications

Following mechanical testing, the pSFDI system was integrated with the biaxial mechanical tester by vertical placement above the testing sample (Figure 1b). Following the procedure of pSFDI-based collagen fiber quantifications, the incident spatial frequency light patterns were produced from an LED projector (Texas Instruments, Dallas, TX, USA), with a wavelength of 490 nm (cyan). A 5-megapixel CCD camera (Basler, Germany) was used to capture the reflected light intensity responses through a rotating linear polarizer (Thorlabs Inc., Newton, NJ, USA), at 37 distinct polarization states (i.e., 0° to 180°, 5° increments).

The above imaging procedure was repeated for three linear phase shifts (0°, 120°, and 240°) of the spatial frequency pattern, based on spatial frequency domain imaging (SFDI) theory [49,50,51]. Image processing and data analyses were completed via custom MATLAB (MathWorks, Natick, MA, USA) programs, to examine the collagen fiber architecture of the tissue’s region of interest (ROI), as defined by the BioRake tines. The quantified CFA information includes the collagen fiber orientation $\theta_{fiber}$ and the degree of optical anisotropy (DOA) at different loading states (i.e., post-preconditioning, and (*T_C_*:*T_R_* = 1:1, 0.5:1, 1:0.1). The DOA stems from the structural anisotropy of the collagen fibers, and therefore, we may attribute the optical anisotropy within the sample to the structural alignment of the CFA (DOA = 1, fully aligned case). Please refer to more details of the pSFDI data analysis in Appendix A, and further information about the pSFDI theory can be found in the recent studies by Goth et al. (2016, 2019) [42,44] and Jett et al. (2020) [45].

**Figure 2:**
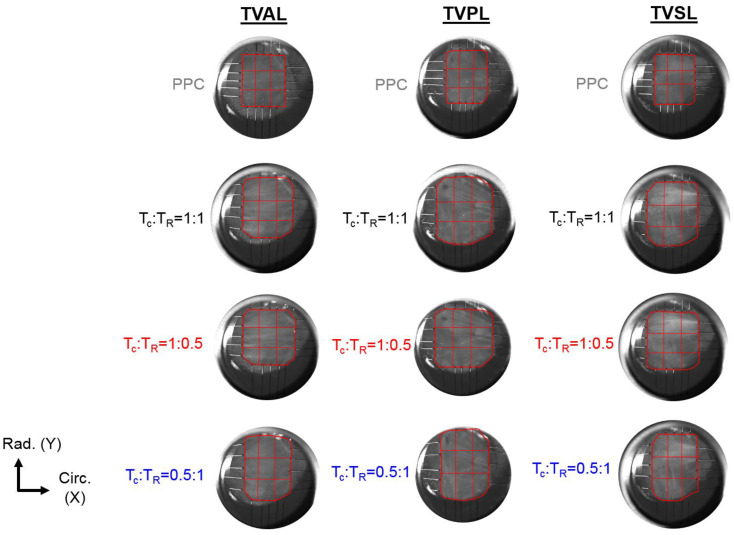
3 × 3 grid sub-regions used for analyzing the spatial variations in the quantified load-dependent fiber orientation angle and the degree of optical anisotropy (DOA) for the anterior leaflet (TVAL), the posterior leaflet (TVPL), and the septal leaflet (TVSL) tissue specimens. (T_C_: circumferential tension, T_R_: radial tension, PPC: post-preconditioning).

In addition to quantifying the load-dependent CFAs of the selected tissue’s ROI, a 3 × 3 grid array as shown in Figure 2 was used to further analyze the spatial variations of the changes in the collagen fiber architecture in response to mechanical loads.

### 2.4. Histological Analysis

To assess the distribution of extracellular matrix (ECM) constituents within each TV leaflet tissue, three rectangular samples (1 × 4 mm) were excised from each of the tested leaflets, with Region 1 defined as the strip near the TV annulus, and Region 3 defined as the strip close to the free edge (Figure 1d). Dissected tissue strips were fixed in 10% formalin at room temperature (23 °C) for 48 h, embedded in paraffin wax, and sectioned (5–7 μm) for histological staining with Movat’s Pentachrome. For all stained tissue samples, three microscopic images were acquired at a 10× objective lens (Olympus, Shinjuku, Tokyo, Japan), and the images were then analyzed using a color deconvolution plugin [52] in ImageJ (National Institute of Health, Bethesda, MD, USA).

In brief, the color deconvolution method was used to separate the red/green/blue (RGB) images into various ECM components, based upon the Movat’s Pentachrome stain: (i) collagen fiber (yellow), (ii) elastin (dark purple), and (iii) non-fibrous ground substance (blue). The staining of these ECM constituents allowed for the determination of the four morphologically distinct layers. The atrialis and ventricularis layers were identified by their outward surfaces towards the atrial and ventricular chambers. The spongiosa can be distinguished from the atrialis layer by the presence of hydrated GAGs and PGs (blue), whereas the fibrosa consists of primarily circumferentially-oriented collagen fibers (yellow). The ventricularis was then defined by the presence of both elastin (dark purple) and collagen (yellow), located below the collagen-rich fibrosa layer [9,10]. The RGB-separated images were next made binary, and thresholding was applied to determine the integrated optical density (*IOD*) of each constituent. Mass fractions ($\omega_{i}$) of the three morphological components were calculated using $\omega_{i}={\mathrm{IOD}}_{i}/\left({\mathrm{IOD}}_{g}+{\mathrm{IOD}}_{c}+{\mathrm{IOD}}_{e}\right)$, where the subscripts g, c, and e denote the non-fibrous ground substance, collagen fibers, and elastin, respectively, and the subscript i signifies the intact tissue, which carries g, c, and e. Additionally, the thicknesses of the whole leaflet and the different tissue layers (i.e., atrialis, spongiosa, fibrosa, and ventricularis, from the atrial to ventricular sides) were measured from each image at three random locations and reported as the mean.

### 2.5. Statistical Analysis

Statistical analysis was performed in Prism (GraphPad, San Diego, CA, USA) for the TV leaflets of the representative heart #1. Firstly, the mass fraction for the three regionally-varying tissue strips were compared within the same leaflet for each of the three TV leaflets. Secondly, the variations in the mass fractions for the different tissue strips in the same relative locations across different leaflets were compared. These observations were compared based on the null hypothesis that the mass fractions for all constituents are uniform across each TV leaflet and show no variance between any two TV leaflets. The two-way analysis of variance (ANOVA) was performed, and *p*-values < 0.05 were considered as statistically significant, pointing towards constituent variations.

## 3. Results

### 3.1. Biaxial Mechanical Testing Results

**Figure 3:**
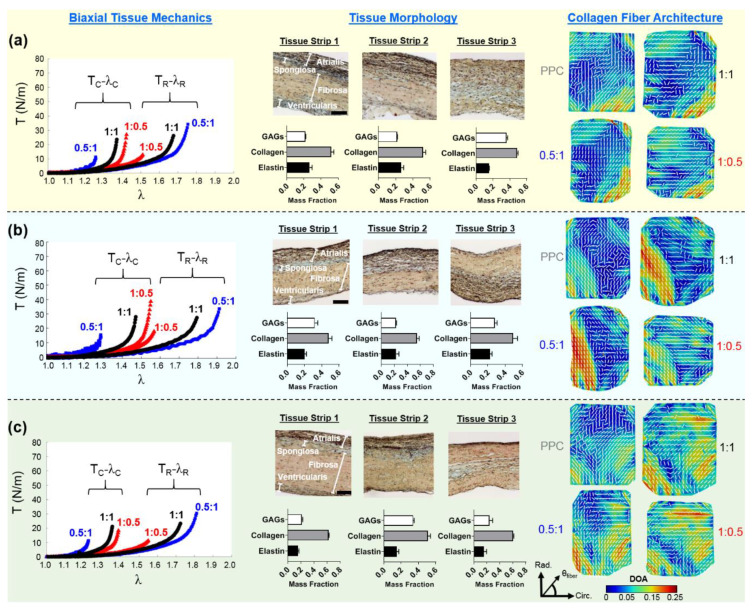
Representative heart #1 quantifications, as follows: (**left**) membrane tension (T) versus tissue stretch (λ) results of biaxial testing experiments (subscripts C and R stand for the circumferential and radial directions, respectively), (**middle**) histology-based evaluations of tissue’s morphology and constituents, and (**right**) the pSFDI-quantified collagen fiber architecture for: (**a**) the TVAL, (**b**) the TVPL, and (**c**) the TVSL specimens. In the right column, the dash lines represent the predicted fiber orientation angle and the colormaps denote the DOA.

J-shape, nonlinear membrane tension-stretch curves were observed for each TV leaflet, in both the circumferential and radial tissue directions (see the left column in Figure 3a–c). Under low tensions (0–2 N/m), there was a relatively linear and compliant response, where minor increases in the applied loading caused substantial increases in the tissue stretches (Figure 3a–c). This low-force regime of the membrane tension-stretch curve contrasted with the stiffer, nearly asymptotic portion of the mechanical behaviors observed under larger applied loads (>10 N/m). For three TV leaflets from the representative heart #1, the tissue stretches changed minimally between the two equibiaxial loading states ($T_{C}:T_{R}=$ 1:1 and 2:2), yielding an increase of 2.09% in the circumferential stretch $\lambda_{C}$ and a 4.43% increase in the radial stretch $\lambda_{R}$, with those changes as the average over the TVAL, TVPL, and TVSL specimens (Table 1). Furthermore, under non-equibiaxial loading ($T_{C}:T_{R}=$ 1:0.5 and $T_{C}:T_{R}=$ 0.5:1), the investigated TV leaflets exhibited a larger stretch in the radial direction, regardless of the direction with the dominant load. For example, in the TVAL specimen, a circumferential stretch $\lambda_{C}$ of 1.42 was observed, together with a radial stretch $\lambda_{R}$ of 1.51 under the circumferentially-dominant loading (i.e., $T_{C}:T_{R}=$ 1:0.5). Interestingly, the TVPL specimen displayed the most compliant mechanical behaviors among all the three TV leaflets in both the tissue’s circumferential and radial directions (Table 1 and Figure 3).

The mean ± standard error of the mean (SEM) values of the tissue stretches from the three porcine hearts (*n* = 3) were determined and reported as follows: (i) greater extensibility in the radial direction under equibiaxial loading (i.e., $T_{C}:T_{R}=$ 1:1, TVAL: $\lambda_{C}$ = 1.35 ± 0.03, $\lambda_{R}$ = 1.59 ± 0.06; TVPL: $\lambda_{C}$ = 1.43 ± 0.02, $\lambda_{R}$ = 1.71 ± 0.03; TVSL: $\lambda_{C}$ = 1.39 ± 0.01, $\lambda_{R}$ = 1.77 ± 0.04); (ii) smaller changes in the circumferential extensibility under circumferentially-dominant loading (i.e., $T_{C}:T_{R}=$ 1:0.5), compared to the radial direction (i.e., TVAL: $\lambda_{C}$ = 1.35 ± 0.03, $\lambda_{R}$ = 1.52 ± 0.01; TVPL: $\lambda_{C}$ = 1.49 ± 0.02, $\lambda_{R}$ = 1.55 ± 0.01; TVSL: $\lambda_{C}$ = 1.43 ± 0.02, $\lambda_{R}$ = 1.61 ± 0.05); (iii) under radially-dominant loading (i.e., $T_{C}:T_{R}=$0.5:1), the TVSL displaying the greatest extensibility among the TV leaflets (TVAL: $\lambda_{C}$ = 1.25 ± 0.02, $\lambda_{R}$ = 1.67 ± 0.05; TVPL: $\lambda_{C}$ = 1.28 ± 0.01, $\lambda_{R}$ = 1.81 ± 0.05; TVSL: $\lambda_{C}$ = 1.24 ± 0.01, $\lambda_{R}$ = 1.87 ± 0.04). The greater equibiaxial loading protocol (i.e., $T_{C}:T_{R}=$ 2:2) was only used for TV leaflets extracted from heart #1, based on the observed minimal changes between protocol $T_{C}:T_{R}=$ 2:2 and protocol $T_{C}:T_{R}=$ 1:1.

### 3.2. Histological Results

The thicknesses and percent mass compositions for each tissue layer of the TV leaflets, as found by histological analysis, are summarized in Table 2. The TVSL was shown to be the thickest leaflet across all three hearts (783.1 ± 62.6 µm), while also containing the largest percent mass composition for the ventricularis layer (12.5%), and the greatest mean total thickness for the fibrosa (363.3 ± 41.1 µm). The TVPL presented the greatest percent mass composition for both the atrialis and the fibrosa layers (A = 33.3%; F = 50.0%) among all leaflets, with the second largest overall mean total thickness (705.4 ± 22.2 µm). Finally, the smallest mean total thickness across all leaflets occurred in the TVAL (614.0 ± 23.9 µm). Quantifications of the TV leaflets’ morphological constituents from the representative heart #1 showed that all three TV leaflets are composed primarily of collagen fibers (see the middle column in Figure 3a–c).

First of all, by comparing the different regions within the same TV leaflet tissue (Table 3), significant regional variance in the elastin was noted between Regions 2 and 3 of the TVAL (*p* = 0.023). For the TVAL, the GAGs were also found to vary significantly between Region 1 and Region 3 (*p* = 0.002), and between Regions 2 and 3 (*p* = 0.001). For the TVPL, significant differences were found in the GAG contents between Regions 1 and 2 (*p* = 0.025). In contrast, the TVSL exhibited significant differences in both the collagen (*p* = 0.029) and the GAGs (*p* = 0.004) between Region 1 and Region 2, while only varying in the GAG contents between Regions 2 and 3 (*p* = 0.017).

Secondly, by comparing similar regional tissue strips across all TV leaflets from heart #1 (Table 4), the TVAL and the TVPL deviated significantly only in the GAG contents for Region 1 (*p* = 0.029), whereas the TVAL and the TVSL differed significantly, in both the elastin (*p* = 0.018) and the GAGs (*p* = 0.004) for Region 1, and in the collagen (*p* = 0.019) and the GAGs (*p* = 0.004) for Region 3. The TVSL showed significant differences when compared to TVPL in all the 3 regions, primarily for the collagen content (*p* = 0.006) and the GAG contents (*p* = 0.016) in Region 1, for the GAG contents (*p* = 0.007) in Region 2, and for the collagen content (*p* = 0.034) in Region 3.

### 3.3. Load-Dependent Collagen Fiber Architecure

**Figure 4:**
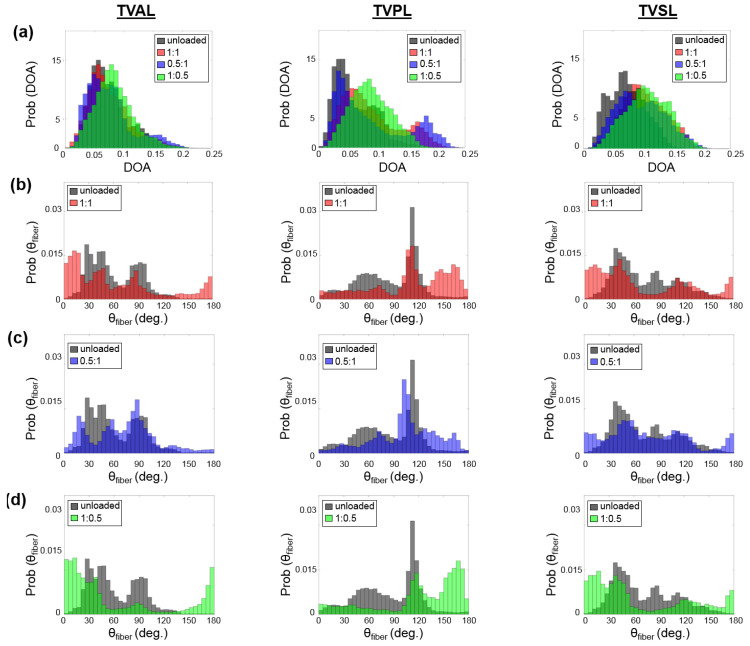
Comparisons of the load-dependent collagen fiber architecture for the entire tissue’s ROI described in Table 1. (see the right column in Figure 3a–c): (**a**) the predicted DOA, (**b**) the predicted $\theta_{fiber}\;$ between unloaded and equibiaxial loading ($T_{C}:T_{R}=$ 1:1), (**c**) the predicted $\theta_{fiber}$ between unloaded and radially-dominate loading ($T_{C}:T_{R}=$ 0.5:1), and (**d**) the predicted $\theta_{fiber\;}$ between unloaded and circumferentially-dominated loading ($T_{C}:T_{R}=$ 1:0.5).

**Figure 5:**
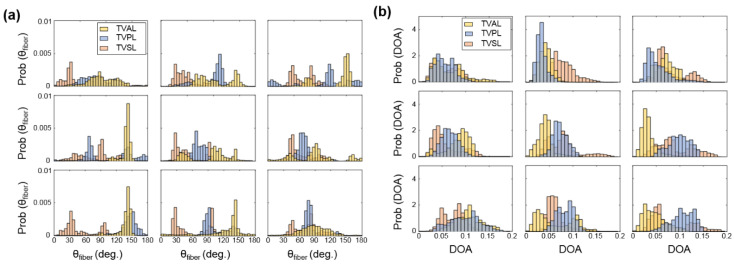
3 × 3 grid comparisons of (**a**) the predicted $\theta_{fiber}$, and (**b**) the predicted DOA in the unloaded state for the TVAL, TVPL, and TVSL specimens from heart #1.

For each TV leaflet, the load-dependent changes in both the DOA and the collagen fiber orientation were quantified throughout the entire tissue’s region of interest (see the right column in Figure 3a–c), with the representative specimen’s histograms presented in Figure 4, whereas the results of the regional analysis, based on the 3 × 3 grid array, are shown in Figure 5, at the unloaded state, to highlight the TV leaflets’ intrinsic characteristics. Furthermore, regional analysis regarding varying biaxial loading protocols can be found in Appendix B.

Four primary trends were observed in the quantification of the collagen fiber architecture of the three representative TV leaflets from heart #1, with an attempt to relate the collagen fiber spatial alignment and reorientation to the direction of applied loading.

**Figure A2:**
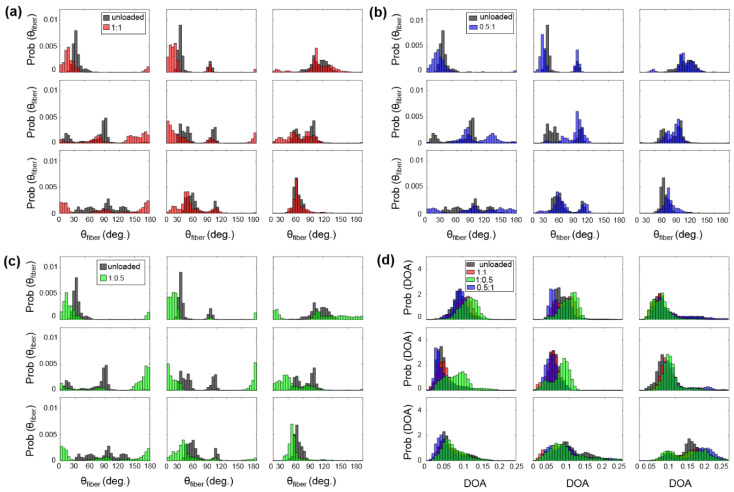
3 × 3 grid comparisons of the predicted $\theta_{fiber\;}$: (**a**) between unloaded and equibiaxial loading, (**b**) between unloaded and radially-dominate loading, and (**c**) between unloaded and circumferentially-dominate loading. (**d**) Comparison of the predicted DOA among all the states for the TVAL specimen of heart #1.

**Figure A3:**
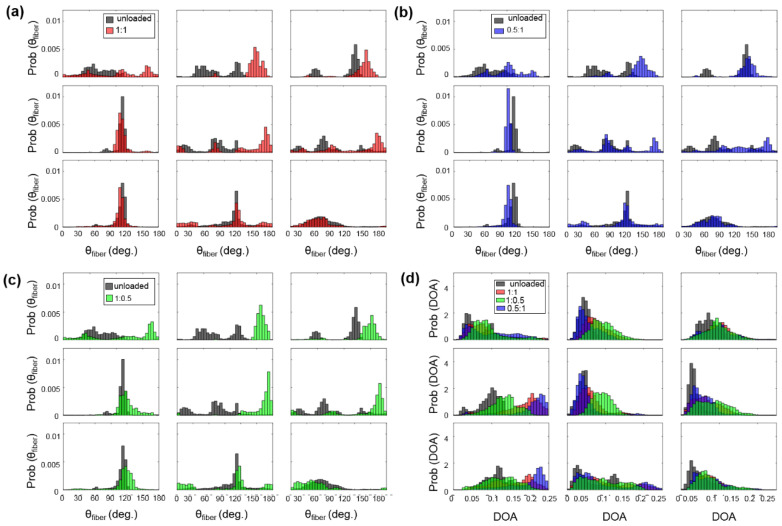
3 × 3 grid comparisons of the predicted $\theta_{fiber\;}$: (**a**) between unloaded and equibiaxial loading, (**b**) between unloaded and radially-dominate loading, and (**c**) between unloaded and circumferentially-dominate loading. (**d**) Comparison of the predicted DOA among all the states for the TVPL specimen of heart #1.

**Figure A4:**
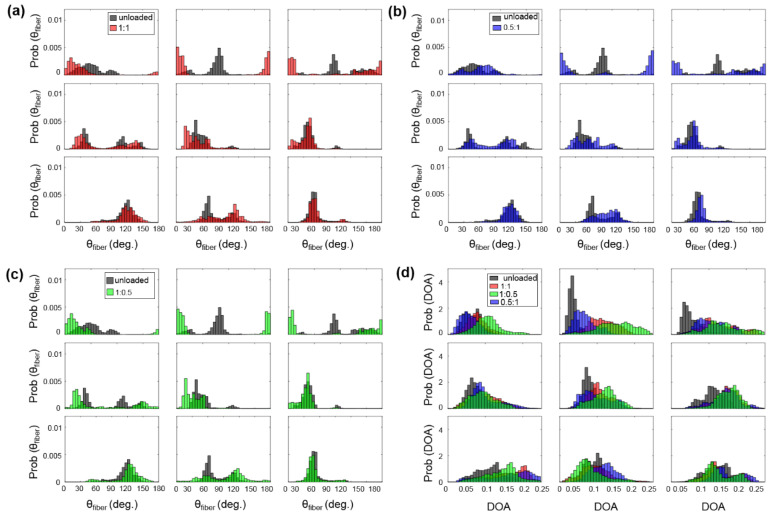
3 × 3 grid comparisons of the predicted $\theta_{fiber\;}$: (**a**) between unloaded and equibiaxial loading, (**b**) between unloaded and radially-dominate loading, and (**c**) between unloaded and circumferentially-dominated loading. (**d**) Comparison of the predicted DOA among all the states for the TVSL specimen of heart #1.

- Firstly, for the non-equibiaxial loading protocols, the collagen fiber orientations $\theta_{fiber}$ displayed a shift towards the direction of the maximum applied loading (Figure 4). Moreover, collagen fiber networks became more aligned, as evidenced by the increasing DOA within the central regions of the tissues (Figure A2, Figure A3 and Figure A4).
- Secondly, the largest changes in the mean collagen fiber orientation $\theta_{fiber}$ were observed in the TVPL under equibiaxial and radially-dominant loading conditions (Table 5), with differences of 37.5% ($T_{C}:T_{R}=$ 1:1), 36.0% ($T_{C}:T_{R}=$ 2:2), and 42.2% ($T_{C}:T_{R}=$ 0.5:1), compared to the post-preconditioning (PPC) state, also referred to as the “unloaded” state in the remaining discussion.
- Thirdly, the largest percent change in the quantified DOA, as compared to the PPC state, occurred under the radially-dominant loading ($T_{C}:T_{R}=$ 0.5:1), with a 10.8% increase for the TVAL, a 39.1% increase for the TVPL, and a 47.4% increase for the TVSL, respectively (Table 5). Under circumferentially-dominant loading ($T_{C}:T_{R}=$ 1:0.5), a minimal change of 1.4% in DOA was seen for the TVAL, with a 27.3% and a 32.4% increase for the TVPL and TVSL, respectively (Table 5).
- Fourthly, across all the loading protocols, the TVSL displayed the largest increase in the DOA (47.3%) under the radially-dominant loading, whereas the smallest change was found for the TVAL (1.4%), under equibiaxial and circumferentially-dominant loading (Table 5).

Moreover, the quantified load-dependent changes of the CFAs for the three TV leaflets of the representative heart #1, considering various biaxial loading protocols, are further elaborated in the following subsections. Further results for heart #2 and heart #3 can be found in Appendix C.

#### 3.3.1. Changes in the CFA Associated with the Equibiaxial Loading States

The TVAL specimen displayed minimal changes in $\theta_{fiber}$ from 61.6° in the PPC state to 60.6° and 60.1° under $T_{C}:T_{R}=$ 1:1 and $T_{C}:T_{R}=$ 2:2, respectively. Similar trends were observed for the spatial alignment of the fibers (i.e., DOA), varying from 0.074 in the PPC state to 0.075 under $T_{C}:T_{R}=$ 1:1 and to 0.072 under $T_{C}:T_{R}=$ 2:2. In contrast, the TVPL showed profound changes in $\theta_{fiber}$, yielding a 30.8% difference from the PPC state (85.9°) to the $T_{C}:T_{R}=$ 1:1 loading protocol (112.4°) and a 29.3% difference when compared to the $T_{C}:T_{R}=$ 2:2 loading state (111.1°). Similarly, the quantified DOA for the PPC state displayed increases of 37.5% and 36.0% compared to $T_{C}:T_{R}=$ 1:1 and $T_{C}:T_{R}=$ 2:2 loading protocols, respectively. For the TVSL, $\theta_{fiber}$ varied from the PPC state (70.9°), by 5.5% for the $T_{C}:T_{R}=$ 1:1 state (67.2°) and 5.8% for the $T_{C}:T_{R}=$ 2:2 state (66.8°), whereas the DOA increased by 40.5% and 37.8% under $T_{C}:T_{R}=$ 1:1 and $T_{C}:T_{R}$ = 2:2, respectively, when compared to the PPC state.

#### 3.3.2. Changes in the CFA Associated with the Circumferentially-Dominant Loading ($T_{C}:T_{R}=$ 1:0.5)

For all TV leaflets, $\theta_{fiber}$ shifted towards the tissues’ circumferential direction with an increased DOA under circumferentially-dominant loading (Figure 3a–c). Specifically, the TVAL displayed a 13.1% difference in $\theta_{fiber}$ between the PPC state (61.6°) and the circumferentially-loaded state (69.7°), together with a negligible change in the DOA (unloaded = 0.074 versus loaded = 0.073). For the TVPL, a 20.3% change in $\theta_{fiber}$ was observed, along with a 27.3% increase in the DOA, whereas the TVSL displayed a shift in $\theta_{fiber}$, from 70.9° (PPC) to 77.1° (circumferentially-dominant loaded) and a greater change in the quantified DOA (32.4%).

#### 3.3.3. Changes in the CFA Associated with the Radially-Dominant Loading ($T_{C}:T_{R}$ = 0.5:1)

When radially-dominant loading was considered, the CFAs of the TV leaflet specimens tended to reorient towards the tissues’ radial direction, with greater changes in the quantified DOAs throughout the tissue compared to other loading protocols. Specifically, the TVAL showed a minimal change in $\theta_{fiber}$ under radially-dominant loading conditions, but a 10.8% increase in the DOA was observed between the PPC (0.074) and radially-dominant loaded states (0.082). In contrast, the TVPL’s collagen fibers reoriented towards the radial direction (122.3°), with a 42.4% change between the PPC and loaded state, while the DOA increased by 39.1%. For the TVSL, the quantified $\theta_{fiber}$ varied from 70.9° (PPC) to 63.6° (loaded), together with a 47.4% increase in the quantified DOAs.

## 4. Discussion

### 4.1. Mechanics-Related Observations

The nonlinear stress-strain response for each TV leaflet was consistent with the findings from the previous studies [14,17,53]. In the present study, for all three TV leaflets, the radial direction of the tissues displayed greater stretches than that of the circumferential direction, under both equibiaxial and non-equibiaxial tensions. This anisotropic tissue mechanical response has also been observed by Pokutta-Paskaleva et al. (2019) [19] and Mathur et al. (2019) [15], through the similar biaxial testing method, among others [14,54]. Essential behaviors of soft collagenous tissues, such as heart valve leaflets, have been further characterized through various fiber reorientation theories and rheological constitutive models. Lanir et al. (1979, 1983) [55,56] and Fung et al. (1984) [57] have contributed to these models for various fibrous soft tissues, taking into consideration the structure of the tissues’ constituents and their mechanical properties, yet, further experimentation is needed to quantify the complex mechanical interactions between constituents that yield this stress-strain response. The anisotropic responses of the TV leaflets, although well documented, have not yet been examined within the context of the pSFDI-based collagen microstructural quantification, as shown in the current pilot study. Supplementing these well-known biomechanical trends within the TV leaflets, together with such novel microstructural quantifications, will ultimately lead to an improved understanding of collagen fiber alignment and reorientation in response to mechanical loading.

### 4.2. Collagen Fiber Architecture-Related Observations

We found in this pilot study that the CFA of each TV leaflet tissue is related to the directional-dependence of the tissue-level mechanics. As previously discussed, each tissue showed a greater extensibility in the radial direction (Table 1), which may be correlated to the collagen fibers’ initial circumferential orientations (see the right column in Figure 3a–c). As increasing tension was applied to the tissue, the asymptotic regime of the membrane tension-stretch curve is associated with the rotation of crimped collagen fibers towards the radial direction. These fibers were then straightened, exhibiting the low-force linear regime of the curve. The collagen fibers’ ability to reorient in response to the applied loading was also shown to result in the higher spatial alignment. This increased alignment of the collagen fibers is exemplified under the radially-dominant loading conditions, where the largest changes in DOA values were found in comparison with the PPC (i.e., unloaded) state (Table 5).

Similar trends in the load-dependent changes in the CFAs were also observed under non-equiaxial loading. Specifically, for all three TV leaflets, the collagen fibers were found to shift towards the direction of dominant loading (see the right column in Figure 3a–c), yielding an increase in the quantified DOAs after fiber reorientation. In contrast, when circumferentially-dominant loading was considered, the quantified $\theta_{fiber}$ became more closely aligned with the tissues’ circumferential directions (Figure 4). These observations in our pilot study are in a good agreement with those findings from the previous studies on the other heart valve leaflets and the TV leaflets with chemical fixation [58,59]. By employing our combined experimental approach, the collagen fiber architecture of the same TV leaflet can be quantified at different equibiaxial and non-equibiaxial loading states, without the use of chemical fixation. This improvement on the previous collagen microstructural observations [37,41] permits further investigations into how the constituent compositions of each TV leaflet tissue contributes to the respective mechanical-microstructure responses under varying loads.

Apart from examining the load-dependent changes among the CFAs for all three TV leaflets, each specimen was also observed in the “unloaded”, or PPC state. The ROI for each TV leaflet was divided into 9 sub-regions (Figure 2), and the collagen fiber orientation and DOA of each sub-region was extracted to further investigate the intrinsic characteristics of the TV leaflets. The TVAL and TVSL leaflets each displayed primarily circumferentially-dominant CFAs throughout all sub-regions of the tissue, with some variation in the radially-oriented collagen fibers of the tissues in the belly portion and near the annulus (Figure 5). The TVPL, on the other hand, exhibited a predominantly radially-oriented CFA throughout the entirety of the tissue, with $\theta_{fiber}$ closely aligned with 90°. The DOA of the TV leaflets remained consistent throughout each section of the tissue (Figure 5), with no statistically significant changes in spatial alignment between the collagen fibers. This assertion can be connected to the collagen fibers’ naturally crimped state, where with greater load, the CFA will straighten and recruit multiple fiber families to compensate for the induced stress on the tissue. Observing the natural CFA and spatial alignment in TV leaflets can be used to further explain the mechanics-microstructure relationship, that has been shown to vary regionally across TV anterior leaflet specimens [36].

### 4.3. Study Limitations and Future Work

Throughout this pilot study, we encountered difficulty in determining an average thickness for each sample, primarily due to non-uniformity and surface imperfections of the tissues, by using a contact-based caliper measurement technique. This limitation was moderately alleviated by measuring the thickness across the leaflet in three different regions and averaging the observed thicknesses. Another limitation of this pilot study is the tine-based biaxial loading technique used. When observing the collagen fiber microstructure via the pSFDI-based technique, the tines inserted in the tissue were captured in the images associated with each phase shift, producing edge artifacts on the CFA map (e.g., see the right column in Figure 3a–c). An extension of this limitation is the displacement-controlled biaxial testing methods used to emulate the target membrane tensions similar to the force-controlled biaxial mechanical tests. This was done by performing displacement-controlled loading to the specimen size associated with the peak loading stress, but due to the stress relaxation of the tissue when determining the peak specimen size, there was a reduction in the target stress values. In regards to the histological limitations presented in this pilot study, we attempted to excise the 3 tissue strips within the observed ROI, but our calculations did not account for the portions of the tissue outsides of the ROI, which may potentially alter the observed trends. Another limitation of this work is the examination of only three representative porcine hearts (*n* = 3). Definite conclusions and trends cannot be drawn regarding the TV leaflets without including a much larger sample size.

Despite these restrictions, our pilot study provides a first look into the investigations of the mechanical-microstructural relationships within TV leaflets. Subsequent studies may warrant a more in-depth examination of the TV with a larger sample size, to draw statistically supported conclusions. Regarding other potential future extensions, the microstructural-mechanical relationship in the collagenous heart valve leaflet tissue can also be examined under various mechanical tests to quantify behaviors such as the preconditioning effect, the stress-relaxation effect, and the creep effect, informing fiber kinematic models, such as the previous ones developed for the mitral valve and the aortic valve [33,34,60,61]. This combined experimental approach could also be used to investigate other collagenous tissues, utilizing its unique capability to elucidate the microstructural-mechanical relationship and inform high-fidelity constitutive models.

## 5. Conclusions

In this pilot study, we have presented a novel systematic framework for characterizing collagenous tissues that utilize both the histology-based morphological assessment and the pSFDI modality in conjunction with biaxial mechanical testing, allowing for a direction examination of the interrelationship between tissue mechanics and collagen microstructures in response to mechanical loads. In the case of the TV leaflets, observing these load-dependent microstructural changes will lead to further developments of improved computational models. Such enhanced computational modeling tools could not only aid in a better understanding of TV function as well as its associated diseases, but they could also be used for patient-specific surgical planning and treatment options. Preoperative guidance in assessing the achievability of a successful tricuspid valve repair, thereby avoiding a less desirable valve replacement, would be of great benefit. The application of this experimental approach is readily applicable to other cardiovascular collagenous tissues (e.g., the aortic valve cusps, and the pulmonary valve cusps), to complement the current understanding in the field. The proposed systematic framework also provides new frontiers and understanding within the field of cardiovascular biomechanics, offering a potential to advance the development of novel clinical therapeutics for TV diseases.

## Figures and Tables

**Table 1:** The quantified tissue stretches of the TVAL, TVPL, and TVSL specimens under various biaxial tension protocols, together with the pixels within the tissue’s region of interest (ROI) corresponding to the pSFDI-quantified results of the load-dependent CFA. Values are reported as mean ± SEM (*n* = 3).

$T_{C}:T_{R}$	Circumferential Stretch $\lambda_{C}$			Radial Stretch $\lambda_{R}$			Pixels in the Tissue ROI (pSFDI)		
	TVAL	TVPL	TVSL	TVAL	TVPL	TVSL	TVAL	TVPL	TVSL
PPC	1.00	1.00	1.00	1.00	1.00	1.00	368,508	434,703	377,398
1:1 ^†^	1.35 ± 0.03	1.43 ± 0.02	1.39 ± 0.01	1.59 ± 0.06	1.71 ± 0.03	1.77 ± 0.04	536,726	624,611	541,161
0.5:1	1.25 ± 0.02	1.28 ± 0.01	1.24 ± 0.01	1.67 ± 0.05	1.81 ± 0.05	1.87 ± 0.04	550,747	626,641	533,910
1:0.5	1.35 ± 0.03	1.49 ± 0.02	1.43 ± 0.02	1.52 ± 0.01	1.55 ± 0.01	1.61 ± 0.05	505,182	567,840	545,645
2:2	1.40	1.50	1.38	1.75	1.87	1.81	584,990	697,596	625,324

^†^ Note that the targeted membrane tension is 25 N/m with respect to the equibiaxial loading protocol, i.e., $T_{C}:T_{R}=$ (25 N/m): (25 N/m).

**Table 2:** Histologically quantified thickness and percent composition of the four tissue layers (i.e., atrialis, spongiosa, fibrosa, and ventricularis) for the TVAL, TVPL, and TVSL specimens. Values are reported as mean ± SEM (*n* = 3).

Tissue Layer	Thickness (Respective %)		
	TVAL	TVPL	TVSL
Atrialis (A)	168.8 ± 18.1 µm (27.5%)	235.1 ± 6.3 µm (33.3%)	230.8 ± 16.3 µm (29.5%)
Spongiosa (S)	77.6 ± 12.4 µm (12.6%)	86.2 ± 11.7 µm (12.2%)	90.8 ± 13.8 µm (11.6%)
Fibrosa (F)	297.6 ± 15.6 µm (48.5%)	289.1 ± 15.4 µm (50.0%)	363.3 ± 41.1 µm (46.4%)
Ventricularis (V)	69.7 ± 4.5 µm (11.4%)	73.8 ± 3.2 µm (10.5%)	98.2 ± 14.9 µm (12.5%)
Total Thickness	614.0 ± 23.9 µm	705.4 ± 22.2 µm	783.1 ± 62.6 µm

**Table 3:** Statistical comparisons (*p*-values) of the constituent mass fractions, between any two regions within each of the three representative heart #1 TV leaflets (see Figure 1d).

	Within the TVAL			Within the TVPL			Within the TVSL		
	Collagen	Elastin	GAGs	Collagen	Elastin	GAGs	Collagen	Elastin	GAGs
Region 1 vs. Region 2	0.593	0.377	0.933	0.266	0.720	0.025	0.029	0.983	0.004
Region 1 vs. Region 3	0.189	0.283	0.002	0.937	0.767	0.449	0.889	0.999	0.809
Region 2 vs. Region 3	0.686	0.023	0.001	0.429	0.997	0.244	0.073	0.989	0.017

**Table 4:** Statistical comparisons (*p*-values) of the constituent mass fraction between any two TV leaflets, regarding the three regions for the representative heart #1 (see Figure 1d).

	Within Region 1			Within Region 2			Within Region 3		
	Collagen	Elastin	GAGs	Collagen	Elastin	GAGs	Collagen	Elastin	GAGs
TVAL vs. TVPL	0.166	0.924	0.029	0.769	0.604	0.964	0.963	0.169	0.054
TVAL vs. TVSL	0.253	0.311	0.952	0.981	0.018	0.004	0.019	0.999	0.004
TVPL vs. TVSL	0.006	0.510	0.016	0.659	0.121	0.007	0.034	0.176	0.441

**Table 5:** Mean, skewness, and standard deviation (SD) for the predicted $\theta_{fiber\;}$ and the DOA of the entire tissue’s ROI (see right column in Figure 3a–c) for the TVAL, TVPL, and TVSL specimens from heart #1, considering various biaxial tension loading conditions.

	**DOA (TVAL)**			**DOA (TVPL)**			**DOA (TVSL)**		
$T_{C}:T_{R}$	Mean	Skewness	SD	Mean	Skewness	SD	Mean	Skewness	SD
PPC	0.074	0.873	0.033	0.064	0.708	0.036	0.074	0.314	0.031
1:1	0.075	0.879	0.035	0.089	0.654	0.048	0.104	0.155	0.036
1:0.5	0.073	1.264	0.038	0.088	0.730	0.059	0.098	0.317	0.041
0.5:1	0.082	0.564	0.032	0.089	0.257	0.034	0.109	−0.012	0.036
2:2	0.072	0.930	0.034	0.087	0.700	0.048	0.102	0.142	0.037
	$\theta_{fiber}$ **(TVAL)**			$\theta_{fiber}$ **(TVPL)**			$\theta_{fiber}$ **(TVSL)**		
$T_{C}:T_{R}$	Mean	Skewness	SD	Mean	Skewness	SD	Mean	Skewness	SD
PPC	61.6°	0.588	30.8°	85.9°	−0.377	34.6°	70.9°	0.621	36.3°
1:1	60.6°	0.906	52.3°	112.4°	−0.698	46.3°	67.2°	0.655	53.5°
1:0.5	69.7°	0.348	38.3°	103.3°	−0.462	37.2°	77.1°	0.398	46.9°
0.5:1	61.6°	0.889	63.4°	122.3°	−1.042	49.3°	63.6°	0.799	56.8°
2:2	60.1°	0.928	52.5°	111.1°	−0.665	47.2°	66.8°	0.655	53.6°

**Figure A1:**
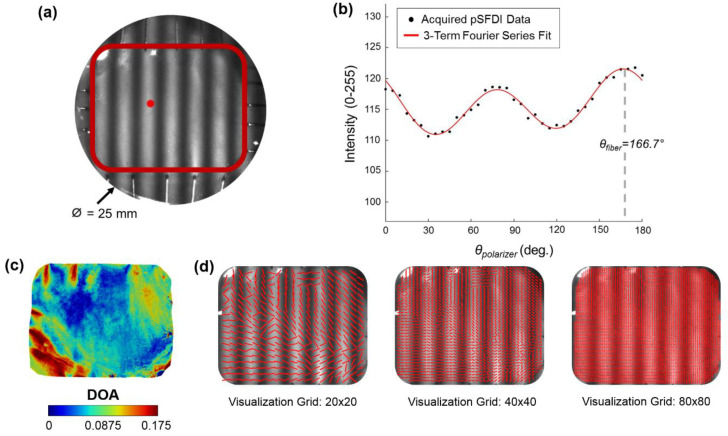
Demonstration of the pSFDI modality to capture the CFA: (**a**) an experimental photo of a representative TV leaflet specimen mounted to the BioTester system, with a defined region of interest as delimited by the red box, and (**b**) the quantified intensity-$\theta_{polarizer}$ relationship of a selected pixel point (see the red dot in (**a**)), together with the fitting of the 3-term Fourier series in Equation (A2) to the acquired data for predicting the collagen fiber orientation angle $\theta_{fiber}$. (**c**) The quantified DOA throughout the ROI, and (**d**) vector plots of the quantified $\theta_{fiber}$ with an increasing level of visualization grid.

## Nomenclature

**Category**	**Abbreviation**	**Definition**
Anatomy	TV	Tricuspid valve
	TVAL	Tricuspid valve anterior leaflet
	TVPL	Tricuspid valve posterior leaflet
	TVSL	Tricuspid valve septal leaflet
	RV	Right ventricle
	FTR	Functional tricuspid regurgitation
	ECM	Extracellular matrix
Tissue Layers	A	Atrialis
	S	Spongiosa
	F	Fibrosa
	V	Ventricularis
Tissue Layer Constituents	CFA	Collagen fiber architecture
	GAGs	Glycosaminoglycans
	PGs	Proteoglycans
Instrumentation	pSFDI	Polarized spatial frequency domain imaging
	SALS	Small angle light scattering
	SHG	Second harmonic generation
	FOV	Field of view
	ROI	Region of interest
Collagen Fiber Quantification	$\theta_{fiber}$	Collagen fiber orientation
	DOA	Degree of optical anisotropy
Mechanics	C	Tissue’s circumferential direction
	R	Tissue’s radial direction
	T	Membrane tension
	PPC	Post-preconditioning
	λ	Tissue stretch

## Appendix A. Quantification of Collagen Fiber Orientation and Degree of Optical Anisotropy

The pSFDI imaging technique combines the ability of co-polarized imaging to quantify the birefringent fiber structures with the depth-discrimination capabilities of SFDI. Interested readers can refer to more details in [44,45,62]. During the polarized spatial frequency domain imaging procedure, polarization-state images (2560 × 2048 pixels) for each phase shift were obtained, and the acquired 37 images were then smoothed via an in-house MATLAB program using convolution with a 5 × 5 uniform kernel. After smoothing, each image was then combined at each pixel to create the predicted birefringent reflected intensity:

(A1) $$I_{out}\;=\;\frac{1}{3}\left(I^{0{}^{\circ}}+I^{120{}^{\circ}}+I^{240{}^{\circ}}\right),$$

where $I^{0{}^{\circ}}$, $I^{120{}^{\circ}}$, and $I^{240{}^{\circ}}$ are the pixel-wise intensities at each respective phase shift. The global maximum for each of these intensity functions occurs when the $\theta_{fiber}\;$ is both perpendicular and parallel with the polarizer’s transmission axis, $\theta_{polarizer}$ (Figure A1).

The quantified birefringent reflected intensity is then approximated by a three-term Fourier cosine series:

(A2) $$\frac{I_{out}}{\tau_{sys}}\;=\;\alpha_{0}+\alpha_{2}\left[2(\theta_{fiber}-\theta_{polarizer})\right]+\alpha_{4}\left[4(\theta_{fiber}-\theta_{polarizer})\right],$$

where $\alpha_{0}$, $\alpha_{2}$, and $\alpha_{4}$ are the three Fourier coefficients and $\tau_{sys}$ is a system dependent coefficient that includes non-birefringent intensity modifiers (Figure A1). The optical anisotropy permits a quantitative examination of the local dispersion of the collagen fibers within the specimen, which can be expressed as the degree of optical anisotropy (DOA) (Figure A2):

(A3) $$DOA\;=\;\frac{\alpha_{2}+\alpha_{4}}{\alpha_{0}+\alpha_{2}+\alpha_{4}}.$$

Please refer to more details about the step-by-step algorithmic procedures in Section 2.3 of [45].

## Appendix B. Spatial Heterogeneity of CFAs

To further investigate the spatial heterogeneity of the collagen fiber architecture for each TV leaflet, a regional analysis was performed, by dividing the tissue’s ROI into 3 × 3 sub-regions (Figure 2). Variations in the fiber orientation angle were observed across different sub-regions of the tissue, as some sub-regions of the tissue showed a significant shift in the fiber orientation angle in response to the mechanical loads, while other sub-regions remained unchanged (Figure A2, Figure A3 and Figure A4).

For example, the TVAL exhibited circumferentially-oriented fibers in the unloaded state, but when radially loaded, the fibers in the center sub-region of the tissue shifted more towards the radial direction compared to the upper portion of the tissue (Figure A2b). The differences in collagen fiber reorientation in response to loading throughout these sub-regions can be connected to the regional variations in biaxial mechanical response previously observed in TV leaflets [36]. The coupling of both the regionally-varied mechanical and microstructural responses can be used to validate affine kinematic theories regarding this relationship. As for the regional variations in the alignment of collagen fibers for each TV leaflet tissue, the belly portion, along with the rough zone (i.e., tissue strip 3, see Figure 1d), showed higher DOA values than the upper portion of the tissue.

To supplement these findings, a histological analysis for each TV leaflet displayed differences in the constituent mass fraction across the tissue domain, further highlighting the potential underlying mechanisms that contribute to collagen fiber reorientation in response to applied loading. The connection between the collagen fiber alignment and the histomorphological compositions has yet to be observed within literature. For example, previous studies are largely limited by optical clearing solutions and a more confined FOV [40,43], and, therefore, regional analyses of the load-dependent CFAs may not be feasible. Our observations, presented in this article, provide unique, spatially-varying collagen fiber architectural information that can be implemented into current rheological models for an accurate representation of the bulk tissue response [55,56,57].

## Appendix C. Quantified Load-Dependent Changes in the CFA for Heart #2 and Heart #3

**Table A1:** The percentage changes in mean, skewness, and standard deviation (SD) for the predicted $\theta_{fiber\;}$ and the DOA of the entire tissue’s ROI for the TVAL, TVPL, and TVSL specimens of heart #2.

	**DOA (TVAL)**			**DOA (TVPL)**			**DOA (TVSL)**		
$T_{C}:T_{R}$	Mean	Skewness	SD	Mean	Skewness	SD	Mean	Skewness	SD
PPC	0.069	0.791	0.025	0.071	1.377	0.029	0.062	0.410	0.026
1:1	−1.6°	12.4°	9.5°	−5.3°	−10.0°	27.0°	14.3°	−23.1°	12.2°
1:0.5	12.2°	−5.2°	41.1°	6.1°	−8.1°	39.9°	43.5°	−47.6°	13.5°
0.5:1	−3.3°	−18.0°	−0.6°	−7.4°	−12.8°	6.7°	15.0°	−16.4°	19.2°
	$\theta_{fiber}$ **(TVAL)**			$\theta_{fiber}$ **(TVPL)**			$\theta_{fiber}$ **(TVSL)**		
$T_{C}:T_{R}$	Mean	Skewness	SD	Mean	Skewness	SD	Mean	Skewness	SD
PPC	93.4°	−0.365	30.0°	105.3°	−0.891	26.3°	106.0°	−0.462	31.9°
1:1	−1.4°	−18.6°	15.2°	6.5°	−19.4°	47.1°	0.3°	27.9°	54.1°
1:0.5	5.5°	12.5°	−2.1°	14.3°	−16.5°	2.6°	11.5°	54.0°	3.4°
0.5:1	−9.9°	−130.9°	25.6°	−3.5°	−46.5°	78.4°	−13.8°	−94.0°	68.9°

**Table A2:** The percent changes in mean, skewness, and standard deviation (SD) for the predicted $\theta_{fiber\;}$ and the DOA of the entire tissue’s ROI, for the TVAL, TVPL, and TVSL specimens of heart #3.

	**DOA (TVAL)**			**DOA (TVPL)**			**DOA (TVSL)**		
$T_{C}:T_{R}$	Mean	Skewness	SD	Mean	Skewness	SD	Mean	Skewness	SD
PPC	0.072	0.861	0.026	0.065	2.774	0.032	0.082	0.515	0.028
1:1	0.3°	−5.8°	20.4°	25.8°	−82.9°	6.6°	4.2°	5.4°	12.0°
1:0.5	12.9°	−35.8°	22.3°	39.5°	−95.8°	12.2°	12.0°	40.2°	15.9°
0.5:1	−7.1°	13.4°	16.2°	12.8°	−86.9°	−1.5°	1.2°	−38.5°	14.9°
	$\theta_{fiber}$ **(TVAL)**			$\theta_{fiber}$ **(TVPL)**			$\theta_{fiber}$ **(TVSL)**		
$T_{C}:T_{R}$	Mean	Skewness	SD	Mean	Skewness	SD	Mean	Skewness	SD
PPC	113.1°	−0.256	22.7°	110.6	−0.948	34.3°	85.0°	0.639	29.4°
1:1	−2.1°	12.9°	35.5°	−5.9°	−35.4°	−5.4°	−5.9°	21.0°	6.2°
1:0.5	−1.1°	−27.9°	12.1°	−3.0°	14.7°	−22.5°	6.9°	−34.2°	−11.7°
0.5:1	−6.7°	59.8°	60.1°	−13.5°	−70.3°	32.6°	−17.7°	98.9°	18.5°
